# Renewing Traditions: A Sensory and Chemical Characterisation of Mexican Pigmented Corn Beers

**DOI:** 10.3390/foods9070886

**Published:** 2020-07-06

**Authors:** Angélica Romero-Medina, Mirna Estarrón-Espinosa, José Ramón Verde-Calvo, Maud Lelièvre-Desmas, Héctor B. Escalona-Buendía

**Affiliations:** 1 Departamento de Biotecnología, Universidad Autónoma Metropolitana, Av. San Rafael Atlixco 186, Col. Vicentina, Mexico City 09340, Mexico; marm@xanum.uam.mx (A.R.-M.); jrvc@xanum.uam.mx (J.R.V.-C.); 2 Unidad de Tecnología Alimentaria, Centro de Investigación y Asistencia en Tecnología y Diseño del Estado de Jalisco. A.C., Camino Arenero 1227, El Bajío, Zapopan 45019, Jalisco, Mexico; mestarron@ciatej.mx; 3 UMR-Transfrontalière 1158 BioEcoAgro, Yncrea Hauts-de-France, Univ. Lille, Univ. Artois, ULCO, UPJV, Univ. Liège, INRAE, F-59000 Lille, France; maud.desmas@outlook.com

**Keywords:** *Zea mays*, Sendechó, volatiles, anthocyanins, HS-SPME, GC-MS, sensory profile

## Abstract

This study was undertaken to explore how the use of pigmented corn as brewing ingredient influences the sensory profile of craft beers, by using both sensory and chemical analyses. Six pigmented corn and barley beers were brewed and then analysed to obtain their sensory characteristics, volatile composition and non-volatile (alcohol, bitterness, anthocyanins and polyphenol content) composition. ANOVAs, Principal Component Analysis (PCA) and Multiple Factor Analysis (MFA) were used to visualise these data for exploring the differences between beers based on the type of malt and to characterise corn beers considering the relationships between their sensory characteristics and their chemical parameters. The sensory attributes such as fermented fruits, cooked vegetables, tortillas, bread, dried fruits and dried chili characterised beers made 100% with pigmented corn. Over 100 volatiles were identified by head space-solid phase micro-extraction coupled with gas chromatography-mass spectrometry (HS-SPME/GC-MS). Among them, phenols and terpenes were the groups of volatiles that better characterised beers containing corn. The content of anthocyanins in corn beers provide the ‘amber-red-cooper’ colours in beers and may prevent the development of off-aromas and tastes. The use of pigmented corn seems to be a good option to renew the traditional ‘Sendechó’ while preserving some of its sensory attributes.

## 1. Introduction

Corn (*Zea mays* L.), a cereal native to Mexico, has been the most important cultivated and domesticated crop from ancient times until today [1]. It comes in a great variety of pigmented grains, with colours that range from white and yellow to purple, red, blue and even black [2,3]. In Mexico, ancient civilizations consumed this cereal as the basis of their diet [1]. They developed several fermented beverages based on specific types of pigmented corn, widely referred to as “corn beers” [4,5,6]. 

Sendechó is one of these typical fermented beverages made by the Mazahuas population in the Valley of Mexico, whose method of production is very similar to the beer process. It is produced with regional ingredients such as blue pigmented corn that goes through a malting process and Guajillo chili [6], which is a traditional ingredient in Mexican cuisine [7]. But as for most of the traditional beverages, the consumption of Sendechó has gradually declined due to changes in eating habits and urbanisation. 

In order to rescue this beverage and preserve some of its sensory properties, we transferred its main ingredients (pigmented corns and guajillo chili) to develop a more modern and consumed beverage, e.g., beer. Therefore, we considered that the use of native varieties of pigmented corn from Mexico for brewing purposes will give added value to both corn grains and beer. Moreover, these types of corn could be used as an alternative cereal in the brewing industry.

Beer is defined as a fermented beverage generally made with four main ingredients: water, malt, hop and yeast [8,9,10]. Traditionally, barley malt is the most common cereal used in the brewing process [9,10]. Nowadays, as a result of the increased consumption of craft beers, the use of alternative cereals and non-traditional ingredients in the brewing process has increased [11,12,13,14]. This allow brewers to create new and different beer styles with a variety of innovative sensory characteristics [9,15].

Several studies of beers have focused on the partial replacement of barley using alternative cereals like wheat [11], rice [12] oats [13] and sorghum [14]. While corn has been considered an economical source of starch [9,16], typically used as an adjunct, authors like Diakabana et al. [17] and Eneje et al. [16] have studied the potential of corn (yellow and white varieties) to produce malt for brewing purposes. Furthermore, in a previous work from our research group, Flores-Calderon et al. [5] developed some beer styles using blue corn malt as the main ingredient. Nevertheless, the use of native varieties of pigmented corn from Mexico has not received similar attention to date.

Since the use of pigmented corn malt as a main ingredient is relatively new to the brewing process [4,5], it is essential to understand the influence of this ingredient on both sensory and chemical composition of these types of beers. Considered as one of the most complex features, beer flavour is generally used in the brewing industry to determine the sensory quality of the beverage. Beer flavour, comprising aromas and tastes, is the result of the combination and interaction of a wide diversity of volatile and non-volatile compounds, originating from the raw ingredients and the brewing process [10,18]. Sensory characteristics of beer are deeply influenced by its chemical profile. Volatile compounds play a key role in the overall aromatic profile of beer. In addition, other non-volatile compounds such as anthocyanins and phenolic components have a significant impact on the sensory attributes such as taste, mouthfeel and colour. Altogether, they serve as a quality indicator and have great importance as they might drive the consumer’s acceptance or rejection of this beverage [9]. Although there are many studies regarding sensory and chemical properties of beer [8,11,18,19], little information could be found on beers made with different varieties of corn [4,5]. Moreover, there are no references of the sensory characteristics and volatile compounds of these type of beers.

Thus, in this study we applied both sensory and chemical approaches, combined with an appropriate statistical methodology, to obtain a complete characterisation of beers, and information about those properties that discriminate between samples and explore the associations between the sensory and chemical properties [20,21]. Specifically, the use of multivariate tools like principal component analysis (PCA) and multiple factor analysis (MFA) to analyse sensory and chemical data at the same time can provide a better overview of the sensory characteristics of the ‘pigmented corn beers’ and chemical components (volatiles and non-volatiles) that can be used as indicators of the use of corn malt.

The main objective of this study was to understand how the use of pigmented corn malt influences the chemical composition and sensory characteristics of beers. To this end, we focused on: (1) characterising the sensory properties of beers made with pigmented corn malt, (2) characterising the volatile composition and non-volatile parameters of the beers (3) identifying sensory attributes that could be influenced by the volatiles and non-volatiles parameters and (4) identifying components (sensory, volatiles and non-volatiles) that can be used as indicators of the use of pigmented corn malt.

## 2. Materials and Methods

In this work, six beers were brewed using different proportions of pigmented corn malt and barley malt (Table 1), hops, water and yeast under an Ale fermentation process. The corn malt was obtained by malting two varieties of pigmented corn (red and blue) and two types of commercial barley malt (base and caramel) were used. In addition, Guajillo chili (*Capsicum annuum*) was used as an adjunct to preserve the main ingredients of the typical Sendechó beverage. Thereafter, chemical properties of each beer were determined by analysing volatile composition (VoC), alcohol content (ABV), international bitterness units (IBU), total anthocyanins content (TAC) and total polyphenol content (TPC). Moreover, sensory analysis was performed to assess the attributes of the six beers. Finally, a correlation between chemical and sensory data was made to understand the contribution of corn malt to the beer sensory properties.

### 2.1. Corn Malting Procedure

Two Chalqueño varieties of red and blue pigmented corn were purchased locally in Milpa Alta, Mexico City. Each variety of corn was used for the preparation of corn malt. The two varieties of pigmented corn were manually cleaned to remove impurities and then were subjected to a micro-malting procedure as described in Mexico Patent No. 365,910 [22]. The red and blue corn grains were soaked for 12 to 24 h respectively, after which the grains were germinated for three days. Green malt was dried afterwards in a kiln at 50 °C for two days to obtain the base corn malt.

### 2.2. Beer Formulation and Brewing Process

Based on a mixture design, six beers (Table 1) were produced using different proportions of corn and barley malts and brewed under the same conditions. Two batches of each beer (15 L) were produced in a microbrewery pilot plant (30 L) at Universidad Autonoma Metropolitana. For all beers, mashing, brewing, fermentation and maturation procedures were performed according to the procedure described in Mexico Patent No. 365,910 (2014) [22]. Hops (Saaz, 3–5 α-acids and Magnum, 12–15 α-acids, HopUnion LLC, US) were added during boiling of mash to achieve 30 International Bitterness Units (IBU). Guajillo chili (*Capsicum annuum*) was also added during this step. Fermentation of wort by a dry top-fermenting yeast *Saccharomyces cerevisiae* (Safale US-05, Fermentis, Marcq-en-Baroeul Cedex, France) took place in a 20 L fermentation tank at 15 °C for 10 days. The green beer obtained was conditioned by adding sucrose (2 g/L) and immediately packed in amber bottles (355 mL) where maturation was carried out at 5 ± 1 °C for three months.

### 2.3. Analysis of Non-Volatile Components

#### 2.3.1. Alcohol by Volume (ABV)

The volume of alcohol was determined following the ASBC method for Beer-4B, where beer and distillate were measured gravimetrically [23]. Alcohol content was expressed as percentage of alcohol by volume (ABV) and was determined by measuring the specific gravity of the distillate (at 20 °C) and referring to its value in tables.

#### 2.3.2. International Bitterness Units (IBU)

IBU is a standard system used to quantify and express hop bitterness in beer due to the amount of iso-alpha acids. The higher the value, the greater the level of bitterness due to the hops [24,25]. 

Determination of IBU was estimated following the ASBC method Beer-23A [26]. Aliquots of beer previously degassed were transferred into a 50 mL centrifuge tube and 0.5 mL of 3 M HCl and 10 mL of 2,2,4-trimethylpentane were added. Consequently, samples were shaken and centrifuged at 2500 rpm for 10 min. The absorbance was measured at 275 nm. IBU was obtained by multiplying the absorbance value by a factor of 50. 

#### 2.3.3. Total Anthocyanin Content (TAC)

The pH differential method was used to quantify total anthocyanins content (TAC) [27]. Results were expressed as mg cyanidin-3-glucoside per litter (C3G/L) for beers based on a molar extinction coefficient (ε) of 26,900 M^−1^cm^−1^.

#### 2.3.4. Total Polyphenols Content (TPC)

The Folin-Ciocalteu spectrophotometric method developed by Singleton and Rossi [28] was used for the determination of total polyphenols content (TPC) in the beer samples. The measurement was compared with a standard calibration curve of a gallic acid solution over the range 50–1000 mg/L. Results were expressed as mg of gallic acid equivalents per litre (mg GAE/L).

### 2.4. Analysis of Volatile Compounds (VoC)

The volatile composition of beers was analysed by headspace solid-phase microextraction (HS-SPME) coupled with gas chromatography (GC) with mass spectrometry (MS). The extraction and concentration of the volatile compounds were performed using the HS-SPME technique using a 1-cm-long divinylbenzene/carboxen/polidimethylsiloxane (50/30 μm DVB/CAR/PDMS) fibre (Supelco, Mexico). The DVB/CAR/PDMS fibre is the most appropriate for flavour volatile analysis as it covers a wide range of groups of volatile compounds, as has been proved by Dong et al. [29]; Riu-Aumatell et al. [30]. The fibre was heated at 250 °C for 15 min between each analysis to prevent contamination from previous injections.

For the HS-SPME procedure, 10 mL of degassed content from each beer were enclosed in 20-mL glass vials containing 2 g of NaCl. Vials were sealed with a polyethylene and silicone septum cap. The sample was magnetically stirred for 10 min at 20 °C ± 1 for sample/headspace equilibration. After this period, the fibre was exposed to the headspace for 35 min with oscillation at 45 °C; this temperature was maintained throughout the extraction step using a heated circulating bath.

After the extraction of volatile compounds, the fibre was immediately desorbed into GC injection port at 250 °C for 10 min to ensure total desorption. For each sample, the analysis was undertaken in duplicate, taking one sample of each batch, and the results were averaged. 

The extracted analytes were analysed in a 7890B/5977A GC-MSD chromatographic system (Agilent Technologies, Palo Alto, CA, USA). Elution and separation of compounds were carried out in a HP-5MS capillary column (30 m × 0.25 mm × 0.25 μm film thickness, 19091S-433UI). Splitless mode was operated in the injector, and helium was used as the carrier gas at a flow rate of 1.3 mL/min. Oven temperature was set to 40 °C, held for 3 min, raised to 190 °C with a heating rate of 5 °C/min, then raised 15 °C/min to 250 °C and held for 20 min. In the GC-MS system the rate of gas carrier was 1.3 mL/min for 37.5 min, raised 0.5 mL/min to 1.8 mL/min and held until the end of the run.

The 5977A MSD (Agilent Technologies, Palo Alto, CA, USA) detector was at 250 °C and the quadrupole was operated in the electron-impact mode at 70 eV and in the scan range (*m/z*) from 29 to 300, with an ion source temperature of 230 °C. 

Data was collected with Mass Hunter GC/MS software (B.07.02.1938). Volatiles were identified by comparing their mass spectrum and their retention times with 36 pure commercial standards. Additionally, all identities were confirmed by comparison of their mass spectra with those of the NIST14 MS library database. In addition, linear retention indices (LRI) were determined with reference to a homologous series of aliphatic hydrocarbons and compared with those reported in literature (Table 2). Since one of the aims of the study was to identify whole volatile compounds that characterise each of the six beer samples, no attempts were made to determine the actual concentration of all identified compounds. The chromatographic peak area was used as an approach of the abundance of each volatile compound in beers and was expressed as arbitrary units (peak area counts × 10^6^) (Table 2).

### 2.5. Sensory Analysis

In this study, we obtained the sensory profile of the six beers using a conventional descriptive method based on the quantitative descriptive analysis [31,32]. Thirteen judges (students from the Autonomous Metropolitan University) were screened and selected based on their sensory acuity to identify and differentiate between the samples, and their potential to describe and communicate sensory perceptions [31]. 

Panel members were trained in the descriptive language of beer category [33]. First, the panel was familiarised with a wide range of commercial beers. Then, panellists generated a list of attributes pertaining to appearance, odour (nasal), aroma (retronasal), taste and mouthfeel of the beer samples. The panel reached a consensus definition of the terms best describing the attributes of barley and corn beers. Physical references were given in order to develop a common and unified understanding of each attribute. Sensory attributes together with their definitions and physical references used by the panel are shown in Table 3. The attributes were followed by an “Ap”, “O”, “A”, “T”, “M”, in case these pertained to appearance, odour, aroma, taste or mouthfeel category respectively. Panellist training was accomplished during twelve 1h-working sessions, which involved learning, associating and rating the intensity of the specific beer attributes developed before. Performance of the panel was assessed by measuring its repeatability between sessions, agreement between panellists and consensus, and the discriminative ability of the panel (Supplementary Table S1) [31].

The trained panel subsequently assessed six samples of beers. Three samples of beer were evaluated per session using a balanced sample presentation design. Each beer sample was evaluated in duplicate by each judge. Samples were coded with a randomly selected three-digit number and were presented in monadic form. All samples were kept refrigerated before being served, and 50 mL were presented in a glass at a range of temperature between 5 to 8 °C. A time-out of 5 min between samples was implemented to minimise fatigue, and water and crackers were provided for palate cleansing.

The panellists were instructed to rate the intensity of each attribute using a 15-cm unstructured line scales anchored by “minimum” to “maximum”. The evaluation of the colour attribute was done following the instructions of the ‘Beer Judge Certification Program (BJCP) Color Guide’ [34]. This guide is designed to allow a beer panellist to quickly estimate the colour of a beer sample in Standard Reference Method (SRM) units. The SRM is a numerical scale developed by the American Society of Brewing Chemist (ASBC) to describe beer colour [34]. The scale ranges from 1 SMR (straw) to 40+ SMR (black). All evaluations were performed in an individual sensory evaluation booth equipped with the electronic data-capturing Fizz^®^ system (version 2.5; Biosystems, Courtenon, France), and all sessions were conducted in the Spanish language.

### 2.6. Statistical Analysis

Data relative to the peak chromatographic areas of the identified volatile compounds were reported as the average of the two independent replicates ± standard deviation (six beer samples, each one by duplicate).

Analysis of variance (ANOVA) was performed on the sensory and non-volatile data (ABV, IBU, TAC, TPC) to ascertain significant differences among all six beer samples. A post-hoc Tukey’s test was carried out when a significant difference (*p* < 0.05) was detected among samples. 

To explore the sensory differences among the beer samples a Principal Component Analysis (PCA) with Pearson correlation coefficients was performed on the table beers x attributes (6 rows × 30 columns) containing the mean intensity scores obtained by each beer for each sensory attribute (calculated over the panellist and the repetitions). No rotation option was applied.

Multiple factor analysis (MFA) is a useful statistical method to analyse the similarities and discrepancies between a set of observations explained by data tables of different groups of variables. It can also be used to show correlation between those sets of variables [21,35].

In this study, MFA was conducted on the data matrices of sensory and chemical (volatile and non-volatiles) variables. More specifically the sensory matrix was divided into two matrices of respectively 19 ‘odour-aroma’ variables (14 odour attributes and 5 aroma attributes) and 7 ‘taste-mouthfeel’ variables (3 taste attributes and 4 mouthfeel attributes). The goal of this separation was to provide a better representation of the chemical data contributions on the odour-aroma and taste-mouthfeel attributes.

Therefore, the MFA was computed on four data tables consisting of: 19 odour-aroma attributes, 7 taste-mouthfeel attributes, 121 volatiles and 4 non-volatile parameters. Additionally, attributes namely colour, turbidity, carbonatation and fullness, which are important for beer characterisation but are not directly influenced by volatile components, were used as supplementary (non-active) variables in the analysis.

All statistical analyses were performed using XLSTAT (version 2018.7, XLSTAT-Sensory package, Addinsoft, Paris, France).

## 3. Results and Discussion

### 3.1. Analysis of Non-volatile Parameters 

Results for the non-volatile analyses of beers are shown in Table 4. We can see significant differences (*p* < 0.05) among all samples on every parameter analysed (ABV, IBU, TAC and TPC).

The content of alcohol (ABV) was significantly higher in beers that contained barley malt than in those made only with corn malt. This might be explained as corn has shown a low diastatic power compared to barley [5,9], which leads to wort contained less fermentable sugars and thus, less alcohol content. As the brewing process remained under the same conditions for all beers, it was surprising to find that the bitterness unit (IBU) in beers were significantly different only for the blended beer made of red corn and barley malt (15.72 IBU) and the blue corn beer (14.57 IBU). These beers showed lowest IBU than the rest of beers (ranged between 15.7 to 19.6). The IBU is a measurement of how much iso-α-acids (1 IBU = 1 ppm iso-humulone) is in the final product, but it does not always really tell if a beer is bitter or not [24]. The amount of iso-α-acids in the beer depends on the time and temperature the hops spend in the boiling step [25]. Thus, minor changes in temperature or time the hops are added to the wort could change the amount of iso- α-acids in beer. Additionally, some authors have reported the susceptibility of this method to the interference from other compounds present in beer, such as polyphenols, that absorb light at the wavelength of measurement (275 nm). Therefore, minor contributions from compounds unrelated to bitterness can be detected (oxidised fatty acids), whereas others contributing to bitterness are not detected [36]. Moreover, coloured beers absorb light which directly decrease the emission intensity and result in lower IBU values [37]. Despite limitations, the IBU method is widely used as an indicator of bitterness in quality control [24,25]. 

Beers containing only corn malt showed a higher content of anthocyanins (TAC) than those blended beer made of barley and corn malt. The anthocyanins value for beers made of blue corn and red corn malt varied from 14.6 to 8.84 mg C3G/L respectively. These results are in agreement with Flores-Calderón et al. [5] who assessed different styles of blue corn beer and reported values that ranged from 13.2 to 18.7 mg C3G/L. A significantly higher difference between beers made of blue corn malt than the one made of red corn malt is expected as a greater amount of anthocyanins has been reported in varieties of blue corn than in the red corn variety [38]. Also, as was expected, the beer made of 100% barley malt did not show presence of anthocyanins. Red and blue corn contain anthocyanins, such as pelargonidin-3-glucoside and cyanidin-3-glucoside, which are responsible of the colour of the grains. Additionally, these anthocyanins have been reported to have various biological activities, such as antioxidant, antimicrobial, antimutagenic and anticancer effects [3,38]. Regarding sensory profile, presence of anthocyanins in beer not only has an effect on colour (ranging from amber-red-cooper) but also on taste and mouthfeel as these compounds could contribute with bitterness and astringency attributes. Thus, the presence of anthocyanins in pigmented corn beers could improve the quality of these beverages.

Finally, all the beers showed considerable amounts of total phenolic content (TPC). The main polyphenols present in a typical barley beer are hydroxybenzoic, cinnamic and ferulic acids. Malt is the main source of polyphenol compounds, providing 70 to 80% of them. Also, a small proportion is originated from hops (20–30%), such as α- and β- acids and their isomeric forms [36,38,39]. In beers made of pigmented corn malt, the presence of polyphenols is also expected. Blue and red corn also have shown the presence of phenolic compounds such as cyanidin-3-glucoside and pelargonidin-3-glucoside, respectively. In addition, ferulic acid and *p*-coumaric acid could be found in these varieties of corn [5]. The results showed significant difference between beers. Higher quantities of TPC were found in those beers made of blue corn malt (BC) and the blended beers made of red and blue corn and barley (RCBa, BCBa). The value of polyphenols ranged between 398.5 to 750 mg GAE/L. Other studies have shown similar results for beers made of blue corn (342 to 560 mg GAE/L) [5,16] and traditional beers made of barley malt (152.0 to 339.12 mg GAE/L) [40]. The differences of the total content of polyphenols may be explained by the variation in the quantity and quality of raw material, the brewing process and the storage conditions during ageing. Polyphenols provide beer with bitterness and astringency but also improve its functionality in terms of foamability, oxidative stability and heat stability which help to preserve the beverage during storage and ageing [39,41].

### 3.2. Volatile Composition

One hundred and twenty-one volatile compounds were identified in beer samples by HS-SPME/GC-MS. The chromatographic data of the volatile compounds of each beer is summarised in Table 2. Compounds were classified into 12 groups of which, the most abundant include esters, representing ~29% of the volatiles, followed by alcohols (~20%), terpenes (~15%) and phenols (~6%). These compounds, particularly alcohols and esters have been the most reported volatiles in barley beers [42]. The major volatiles detected in this study were consistent with those of previously published studies [11,18,42,43].

As mentioned before, esters were the largest group found in all beer samples. Esters are the most common compounds in the majority of beers and these volatiles are considered desirable as they act in synergy with other compounds and contribute with most of the pleasant fruity-floral aromas in beer [44,45]. According to our results, it seems that beers made with barley malt contain higher number of esters than the beers made with corn malt (Table 2). For instance, ethyl propanoate, ethyl isobutanoate, ethyl pentanoate, ethyl isohexanoate, ethyl benzoate and isopropyl palmitate were only found in beers made with barley (Ba, BCBa, RCBa). It is well known that the presence of alcohols leads the production of esters [44]. Thus, the presence of a greater number of esters in barley beers could be attributed to their content of alcohols, which are precursors of these compounds. 

Esters such as ethyl acetate, 3-methylbutyl acetate, ethyl hexanoate, phenethyl acetate, ethyl 9-decenoate and ethyl decanoate were found in all samples in higher abundance than the rest of the esters. 

Ethyl octanoate, a product of fermentation by *Saccharomyces* yeast, was detected in all five beers that contain corn malt except in the one made of 100% barley malt. Conversely, octanoic acid was more abundant in the barley beer than in the corn beers, which is consistent with Saerens et al. [45] who found that higher levels of unsaturated fatty acids in beers, like in the corn beer samples, result in a decrease in ethyl ester production. The contrary effect can be seen for ethyl hexanoate and hexanoic acid, where in samples that exhibited a higher peak area of the ester, the presence of the acid seems to be reduced (BCBa and RCBa). 

Alcohols were the second largest group of volatiles found in beers. We identified 24 alcohols and some of them were found in all six beer samples such as ethanol, 2-methyl-1-propanol, 3-methyl-1-butanol, 2-methyl-1-butanol, 2-ethyl-1-hexanol, phenylethyl alcohol and citronellol. These alcohols come mainly from alcoholic fermentation while others such as citronellol and phenylethyl alcohol come from the essential oils of hops. According to Lyu et al. [12] and Dong et al. [29] aromas like sweet alcohol, rough, whiskey, fruity and rose could be attributed to these compounds. 

In addition, some alcohols such as 2-furanmethanol, 4-methyl-1-pentanol, 3-methyl-1-hexanol and iso-geraniol were only found in those beers that contain barley malt (Ba, BCBa and RCBa). Of them, 2-furanmethanol is a product of Maillard reactions that occur during the roasting process of malt, especially in the production of ‘dark’ and ‘caramel’ malts; hence the caramel malt used in Ba, RCBa and BCBa beers could be the source of this volatile [30]. Interestingly, to our knowledge, there are no reports of iso-geraniol in beers. This compound is the result of the partial oxidation of geraniol. It was previously identified in some flowers, fruits (grapes) and the essential oil of lemon, imparting a pleasant rose odour [46]. 

In beers, terpenic compounds are generally derived from the hop essential oils, which are added to the wort during the boiling process. These compounds have been related to pleasant aromas like citrus, floweryand lilac [29,30]. We identified 18 terpenes in the beer samples, most of them have previously been reported in barley beers [30,47]. Only linalool, geraniol and humulene, associated with flower, geranium and wood aromas respectively, were detected in all six samples of beer. In turn, limonene and β-myrcene were found in beers made 100% with corn malt (RC, BC). In addition, these beers (RC, BC) showed more abundance of limonene and linalool than the other samples of beer. Interestingly, δ-cadinol and α-cadinol were found in those beers made with blue corn malt but (BC and BCBa) and 3-methoxy-2-naphthalenol was found only in those that contain barley malt (Ba, BCBa, RCBa). Among these terpenes, limonene have been previously reported in corn starch and corn products [48,49]. 

Seven phenol volatile compounds were identified among the beer samples. These compounds contribute to clove and spice aromas in beers, which are desirable in some Belgian styles (amber and Trappist beers) and wheat beers [50]. For instance, 4-ethyl-2-methoxy-phenol was detected in all beers containing red and/or blue corn malt, but not in barley beer. Buttery and Ling [49] reported that 4-ethyl-2-methoxy-phenol is one of the major components in products like corn tortillas and tortilla chips. Furthermore, 2-methoxy-phenol was found only in beers containing blue corn malt. Even though 4-ethyl-phenol and 2-methoxy-4-vinylphenol were found in all beers, these compounds exhibited a higher peak area in those beers that contain both red and blue corn malt (RBC) than in the other beers. Of those, 4-ethyl-phenol is usually found in beers made of wheat malt. This molecule is formed from the biodegradation of hydroxycinnamic acids, such as ferulic and coumaric acid, during wort boiling. In high concentrations it imparts unpleasant aromas like medicinal, phenolic, clove-like, or smoky. However, in some beer styles such Belgian wheat and German Weizen these aromas are appreciated [51,52]. 2-methoxy-4-vinylphenol and 4-vinylphenol (the precursor of 4-ethyl-phenol) have been reported as major components of sweet corn products such as tortillas [53].

Styrene was the most abundant hydrocarbon found in all beer samples. This compound usually comes from the malt and it derives from the metabolism of cinnamic acid in barley malt by top-fermenting yeast [18]. Its presence in the corn beers is explained as its formation occurs in parallel to the formation of 2-methoxy-4-vinylphenol and 4-vinylphenol. Styrene has been described as a “sweet-smelling colourless fluid” [54]. 

Interestingly, dimethyl sulfide (DMS) exhibited a higher peak area in the beer made 100% with barley malt (Ba), followed by those made with corn malt (BC, RC, RBC). DMS is usually lost during the kilning of malt and the boiling of the wort, however its presence in the beer depends on the type of malt used. This sulphur compound has been reported in barley beers. Its presence is desirable in some styles of beers, like some lagers, while in others is not desirable as it adds sweet corn aroma to the beer [10]. In addition, DMS has been identified as an important contributor to the aroma of corn products [48,49].

Additionally, β-ionone was only found in beers made with blue and red corn malt (BC, RC, BCBa, RCBa) with the exception of RBC. This ketone has been previously reported as potential contributor of hop aroma. It has been identified in tortillas and corn dough [49], in late-hopped and dry-hopped beers [55] and in samples of whiskey made with corn [56].

### 3.3. Descriptive Sensory Analysis 

The sensory panel developed a list of 30 attributes to describe the appearance, odour, taste, aroma and mouthfeel characteristics perceived in all beer samples (Table 3). The panel was asked to be as specific as possible in identifying attributes. Some terms and references were similar to those defined in the “beer flavour wheel”, developed by Meilgaard [33], but others were unique attributes related to the presence of pigmented corn and chili. 

**Figure 1:**
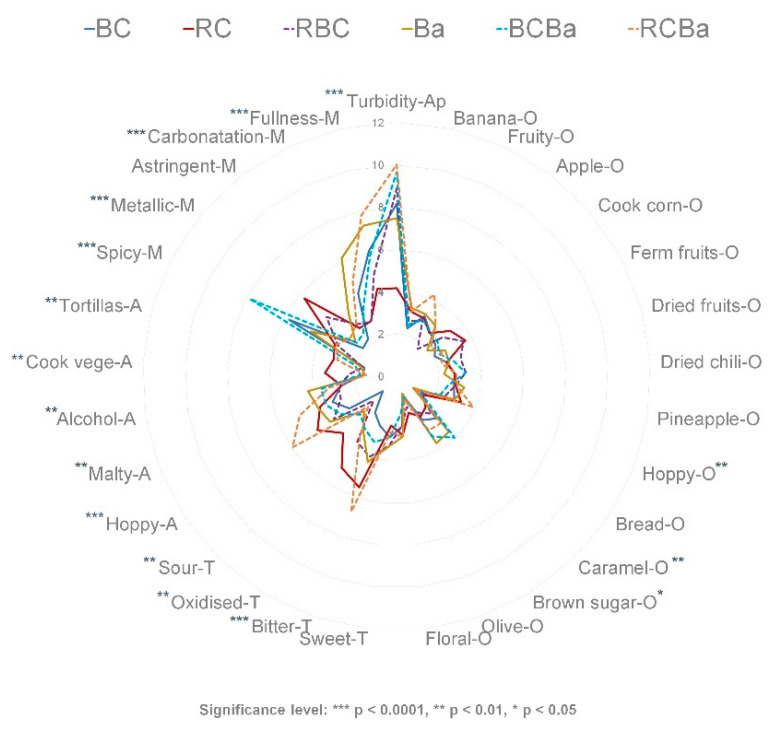
Sensory profile of the six beers. BC = 100% blue corn, RC = 100% red corn, RBC = 50:50 red and blue corn, Ba = 100% barley, BCBa = 50:50 blue corn and barley, RCBa = 50:50 red corn and barley.

The mean scores of the attributes were plotted in a radial diagram (except for the colour attribute) (Figure 1). Significant differences (*p* < 0.05) were found in 17 out the 30 attributes across the samples (Supplementary Table S1). In order to have a complete description of all sensory characteristics of the beers, all attributes were kept and used in the subsequent analysis. We can see that the non-significant attributes were mainly those pertaining to the odour category. These odour characteristics are common to most of the commercial beers and some of them are the result of the volatile compounds developed during the fermentation process (e.g., banana, apple, floral, fruity). Thus, as all steps in the brewing process remained the same, we can expect some similarities between beers.

All beers in this study exhibited a range of sensory characteristics commonly found in most of the commercial beer samples, however some characteristics such as ‘dried fruits-O’, ‘dried-chili-O’, ‘brown sugar-O’, ‘tortillas-A’ and ‘spicy-M’ are not in the common lexicon of beers [33]. Thus, the pigmented corn malt and the chili used in these beers appear to contribute to the development of these attributes. Despite the fact that cooked vegetable-A and cooked corn-O are usually associated with off-aromas in barley beers, we could expect that the pigmented corn beers develop these characteristics as they are sensory attributes found in the ‘Sendechó’ beverage [4] and in many corn-derived products [48,49].

The beer made 100% with barley malt (Ba) had a significantly higher intensity of brown sugar and caramel attributes than the other beers, which was expected as the caramel malt used in this beer contributes with the development of these aromas. Furthermore, alcohol aroma was higher in barley beer (Ba) than in the others, this is reasonable as barley malt contributes more to the formation of fermentable sugars than corn and therefore barley beers had higher alcohol content than the beers made with corn malt (see Table 4).

RC and RCBa, both containing red corn malt, were rated higher in bitter taste, as compared to the other beers. In general, those beers containing red corn malt (RC and RCBa) were characterised by higher intensity of aroma attributes such as cooked vegetables and tortillas, related to the type of corn used. In addition, sour taste, oxidised and metallic sensations were scored high in the RC beer. The latter attributes are usually associated to an ageing effect [32]. 

Despite the fact that Guajillo chili was added to all the beers in the same proportion and conditions during the brewing process, the perception of spicy attribute was different in all the beers. For instance, the beer made of blue corn and barley (BCBa) was rated significantly higher in spicy mouthfeel than the rest of beers, followed by blue corn beer (BC). The perception of the ‘spicy’ or ‘pungent’ sensation elicited by the capsaicin (the active ingredient of the Guajillo chili) may be influenced by factors such as the temperature, acidity and carbonatation of the beverage [57]. In addition, phenolic compounds that evoke an oral irritation [39,41] might increase the perception of this sensation. Thus, the content of polyphenols in BC and BCBa might contribute to the increase perception of the attribute spicy. Beers made with barley (Ba, BCBA, RCBa) had a higher carbonatation sensation than those beers made with pigmented corn malt (BC, RC, RBC). The perception of the fullness, which is associated with the body of the beer, was higher in the beers that contain blue corn and/or barley malts (BC, Ba, RCBa and BCBa) than in the ones made with red corn malt (RC and RCBa). The fullness palate sensation is related to the unfermentable sugars namely dextrins, developed during the mashing process. These compounds contribute to the body of the beer without imparting sweetness [10].

The assessment of a beer’s appearance includes its colour, which according to the SMR colour chart it can range from straw to black. All beer samples analysed are in the range of the colours that goes from 10 SMR to 15 SMR units. Significant difference can be observed (Supplementary Table S1) between the RC beer with a ‘medium amber’ colour (9 SMR), the BC beer with a ‘light brown-reddish’ colour (15 SMR) and the rest of the beers with a ‘cooper-red’ colour (12–13 SMR). It is well known that malt has the greatest impact on beer colour because of its content of melanoidins and Maillard compounds, which add colours that range from yellow, orange to red and brown [58]. In this case, the anthocyanins in the pigmented corn beers contribute to develop of these ‘amber–red-cooper’ colours, especially in those beers made 100% with red and blue corn malt. In acidic solutions such as beer, anthocyanins are chemically stable and turns their colours to reddish tones [3].

**Figure 2:**
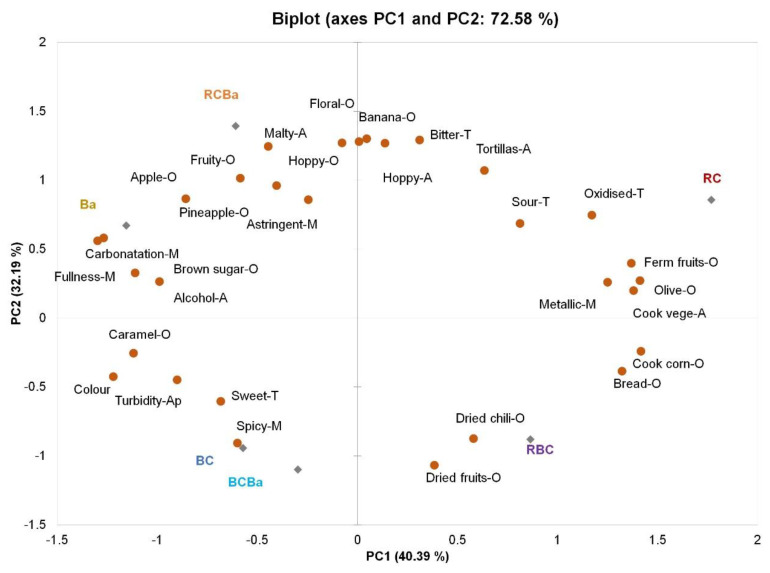
Principal Component Analysis (PCA) bi-plot of variables and individuals of descriptive sensory data. BC = 100% blue corn, RC = 100% red corn, RBC = 50:50 red and blue corn, Ba = 100% barley, BCBa = 50:50 blue corn and barley, RCBa = 50:50 red corn and barley.

With the aim of illustrating the differences among beers produced by different types of malt (red corn, blue corn and barley), a PCA was applied on the total data set of 30 attributes. The biplot obtained is shown in Figure 2. The first two components (PC) explained 72.58% of the total variation in the samples with contributions of 40.39% by PC1 and 32.19% by PC2, where most of the attributes contributed considerably to samples discrimination. 

PCA permitted a clear-cut separation of the samples based on the type of malt used. 

PC1 opposed the beers made with barley malt like Ba, RCBa and BCBa (on the left) to the RC and RBC beer (on the right). On the other hand, PC2 opposed beers made of red corn malt (positive side) to beers made of blue corn malt (negative side). The RC beer was characterised by attributes such as fermented fruits-O, olive-O, tortillas-A, cooked vegetables-A, metallic-M and oxidised-M. On the contrary, BC and BCBa were characterised by spicy-M, sweet-T, Turbidity-Ap.

The beer made of 100% barley malt (Ba) was discriminated along PC1 (at the negative side) and was characterised by brown sugar-O, apple-O, alcohol-A, carbonatation-M and fullness-M. 

Blended beer made of both type of corn malt (RBC) was placed in between red corn beer (RC) and blue corn beer (BC), sharing attributes of both malts used such as bread-O, cooked corn-O and dried chili-O and dried fruits-O. This behaviour was also shown in blended beer made of red corn and barley malt (RCBa), preserving the sensory characteristics of both 100% barley (Ba) and 100% red corn (RC) beers such as apple-A, fruity-A, banana-A, malty-A and floral-A, attributes that are more common in typical barley beers. 

These sensory data showed that by adding corn malt to the beer formulation, the sensory profile of the typical barley beer can be reached easily, while preserving at the same time odours and aromas of corn products, especially those of the Sendechó beverage such as corn and spicy and dried chili [4].

### 3.4. MFA of Sensory Attributes and Chemical Data 

In this study, MFA was used to explore the differences and similarities between beers due to the type of malt used in brewing. In addition, MFA helped to identify associations between sensory and chemical datasets that brought us to know those components (sensory and chemical) that can be used as markers of beers made with pigmented corn malt.

**Figure 3:**
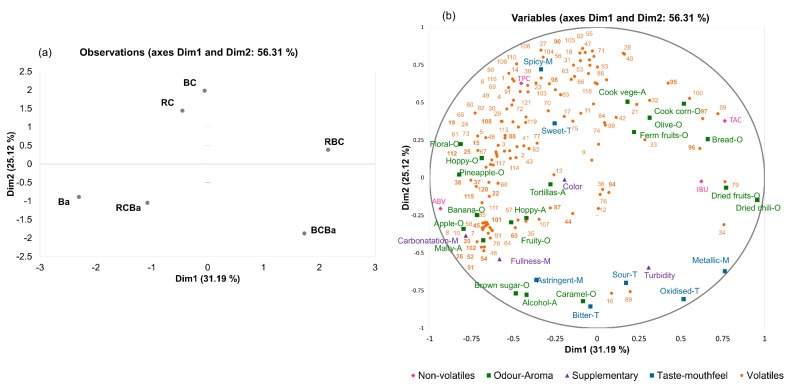
Multiple Factor Analysis (MFA) of descriptive sensory and chemical data of the six beer samples. (**a**) Observations plot of MFA, (**b**) Variables plot of MFA. Numbers correspond to the compounds listened in Table 2. BC = 100% blue corn, RC = 100% red corn, RBC = 50:50 red and blue corn, Ba = 100% barley, BCBa = 50:50 blue corn and barley, RCBa = 50:50 red corn and barley.

The first two dimensions (Dim 1 and Dim 2) in Figure 3 accounted for 56.31% of the total variation with contributions of 31.19% by Dim 1 and 25.12% by Dim 2. 

First, the variable plot (Figure 3b) shows that Dim 1 separates samples based on the sensory ‘odour-aroma’ attributes (in green; 34.05% of the variance) and the ‘non-volatile’ components (in pink; 34.53% of the variance). For Dim 2, the groups of variables ‘volatiles’ (in orange) and ‘taste-mouthfeel’ (in blue) are those that contribute the most to the dimension with 22.41% and 44.91% of variance respectively. The plot of the individuals (Figure 3a) allows us to visualise the global resemblance between beers by considering the information of all variables (sensory and chemical). It clearly showed that Dim 2 separated the samples based on the type of malt used, with beers made with pigmented corn (red and blue) on the top of the plot, and the beers that contain barley malt plotted on the bottom (Figure 3a). 

Second, the RV coefficients (Table 5) show the relationship between the data matrices, the closer the RV coefficient to 1, the more similar the matrices [21,35]. According to the RV, a good correlation can be observed between the ‘odour-aroma’ and ‘non-volatile’ variables (0.740). Moreover, a better correlation between ‘volatiles’ and ‘taste-mouthfeel’ variables (0.649) than for ‘odour-aroma’ and ‘volatiles’ data matrices (0.509).

A deeper analysis of Figure 3 allows detailing these relations between the different types of variables that strengthen the characterisation of the beers. On the negative side of Dim 1 of the variable plot (Figure 3b), it can be observed that the sensory attributes floral-O, hoppy-O and pineapple-O are positively correlated mainly with esters (i.e., ethyl butanoate (48), phenylethyl acetate (61), ethyl (E)-4-decenoate (65), ethyl decanoate (67), isoamyl octanoate (68), terpenes (i.e., geraniol (108), δ-cadinene (112), humulene oxide (115), δ-cadinol (120), and alcohols (i.e., phenylethyl alcohol (15), citronellol (19) and 1-decanol (22)). Numbers correspond to those on Table 2. Esters and alcohols are well known for their floral and fruity contribution to the beers, and terpenes are more likely associated with herb and green odours-aromas, which are consistent with the description of the hoppy odour. These correlations between the sensory attributes and the volatiles compounds strengthen the aromatic profile of the barley beer (Ba). Also compounds such as 2-nonanone (88), heptanoic acid (37), 2-ethylhexanoic acid (38) and acetaldehyde (25) were also correlated with the sensory attributes mentioned before. The positive correlation of these fruity and floral sensory attributes with carboxylic acid compounds could suggest that the presence of esters, even in low levels, might reduce the perception of off-aromas like sweat and rancid, caused by octanoic acid [59]. 

On the positive side of Dim 1, RBC (Figure 3a) can be separated from the other beers mainly by the presence of phenol volatile compounds. Among them, phenol (94), 2-methoxyphenol (95), 4-ethylphenol (96) and 4-ethyl-methoxy-phenol (97) showed association with the sensory attributes related to the presence of pigmented corn malt such as cooked vegetables-A, cooked corn-A, olive-O and fermented fruits-O (Figure 3b). These compounds and the sensory attributes allow us to differentiate between the beers made 100% with corn malt, suggesting that these phenol compounds could be use as indicators of the use of pigmented corn in the brewing process.

On the negative side of Dim 2, we found positive correlations between attributes such as malty-A banana-O, brown sugar-O, tortillas-A and fruity-O and the compounds 2-furanmethanol (8), ethyl propanoate (44), propyl acetate (45), ethyl pentanoate (51), ethyl isohexanoate (52), ethyl hexanoate (54), iso-geraniol (20), acetophenone (87), 2-acetylpyrrol (101) and tetramethyl-pyrazine (102). The presence of these compounds, characterised by fruity, bready, brown sugar and caramel aromas [45,60], is consistent with the use of roasted malts (caramel malt) in the beers associated to these compounds (RCBa and Ba). Furthermore, on Dim2 (negative side), a weak correlation was also found for benzeneacetaldehyde (26) with astringent, which is consistent with the results obtained by Owusu et al. [61], where the presence of this compound has been associated with the astringent mouthfeel in products as cocoa and dark chocolates.

The positive side of Dim 2 is positively correlated with beers made from red corn malt (RC) and blue corn malt (BC) (Figure 3a, top side). These beers are well characterised by compounds such as linalool (106), limonene (105), β-ionone (90) and 4-ethyl-2-methoxy-phenol (90). These volatile compounds have been found in other corn products such as tortillas and pop-corn [49,60] and especially limonene and β-ionone have also been reported in samples of whiskey made with corn [56]. Thus, these compounds could also be used as markers of the presence of corn in beers. 

In addition, the spicy attribute was strongly correlated with 2-methyl-5-(1-methylethyl)-phenol (98) well known as carvacrol -a key aroma compound in oregano spice- that is concordant with the pungent mouthfeel associated with this compound [60]. Unexpectedly, dimethyl sulfide (103) which usually imparts cooked vegetable off-aroma also showed a positive correlation with the spicy attribute. This behaviour could be attributed to the high abundance of phenylethyl alcohol (15) that could suppressed the perception of this compound [8].

Correlations between the non-volatiles variables (ABV, IBU, TPC, TAC) and the sensory and volatile data were also studied. For instance, a positive correlation was found between alcohol sensory attribute and alcohol content (ABV). Regarding the total polyphenol content (TPC), a negative correlation was observed between TPC and metallic and oxidised sensory attributes, confirming that polyphenols help to retard the development of these attributes in beer [54]. Moreover, TPC showed a positive correlation with carvacrol volatile (98). According to Lee et al. [62] carvacrol is a volatile compound that has exhibited potent antioxidant activity.

It is well known that anthocyanins do not impart aromas, but sometimes these compounds have been related to an astringent or bitter taste [41]. Even though, no obvious correlations were found between TAC and bitter or astringent attributes. The results showed a positive correlation between TAC and phenol compounds such as phenol (94), 2-methoxy-phenol (95), 4-ethyl-phenol (96) and 4-ethyl-2-methoxy-phenol. This could suggest an interaction between the anthocyanins that comes from corn malt and those phenol volatile compounds. According to Dufour and Sauvaitre [63] and Ruta and Farcasanu [64], interactions between anthocyanins and some aroma compounds such as phenol and 2-methoxy-phenol, lead the formation of copigments, which improve the stability of the anthocyanins and hence the colour stability of the beverage.

Apparently, no positive correlation was found between IBU parameter and bitter sensory attribute. However, there are other components that could contribute to the perception of bitterness such as the Maillard products formed during the kilning and roasting process of caramel and dark malts [57,65]. In addition, bitterness can be masked by sweetness due to sugars (residual sugar) that remain after the fermentation process. As has been mentioned before, IBU measures a beer’s bitterness due to the α-acids of the hops, which gives an approximate idea of beer bitterness but there are other compounds that could impart or mask the bitter taste. Thus, it is not possible to directly correlate IBU to the perceived sensory bitterness [41]. 

Finally, the different groups of variables (sensory and chemical) had different influences in each beer. The major difference was found for the BCBa and RCB which were mainly described based on taste-mouthfeel attributes and non-volatile parameters respectively. Beers Ba and RCBa were mainly described based on the odour-aroma attributes and volatile compounds. For beers made 100% with pigmented corn (RC and BC) the group of volatiles had more influence in their characterisation. Overall, the volatile composition also separates beers depending on the presence of corn, supporting the fact that the use of corn as an ingredient clearly alters the sensory profile of beers. 

## 4. Conclusions

It is well known that sensory evaluation plays an important role when new products needs to be characterised, but it is also an important quality factor used to control the brewing process. In this study, sensory evaluation enabled the complete description of the corn beers. 

Beers made with these specific types of pigmented corn (red and blue) are mainly characterised by fermented fruits, cooked vegetables odours, tortillas, bread, dried fruits and dried chili. 

We evidenced for the first time that among the groups of volatile compounds, ketone (β-ionone), terpenes (limonene, linalool) and phenol volatiles (2-methoxy-penol, 4-ethyl-phenol and 2-methoxy-4-vinylphenol, 4-ethyl-2-methoxy-phenol, 4-ethyl-2-methoxy-phenol), as well as the presence of anthocyanins appear as relevant criteria for corn beers differentiation. The latter can also be used as indicators to determine whether a beer is made with pigmented corn malt or not and therefore be used as a quality parameter in further studies. Moreover, the study of the relationship between the sensory attributes and the chemical parameters by MFA allowed to elucidate the effect of each type of malt (red corn, blue corn and barley malt) on the chemical parameters (VOC, ABV, IBU, TAC, TPC) and the association with the sensory attributes.

Both varieties of corn malt showed a clear influence in all parameters measured, especially in their sensory profiles. However, the blended beers (RCBa and BCBa) show the closest resemblance to a typical barley beer, while preserving those traditional aromas and tastes of the ‘Sendechó’ beverage. Additionally, the use of pigmented corn malt could help to prevent the development of off-aromas (e.g., oxidised), which could extend the shelf life of the beer.

This study will enable the Mexican brewing industry to gain an insight into the use of alternative and native cereals, which could renew and preserve autochthonal beverages in a modern way. Whether the sensory characteristics of these beers may carry the acceptance or rejection of consumers needs to be further investigated.

## Figures and Tables

**Table 1:** Beer formulations.

Prototype	Abbreviation	Beer Formulation
1	BC	100% Blue corn malt
2	RC	100% Red corn malt
3	RBC	50% Red corn malt, 50% blue corn malt
4	Ba	85% Barley base malt, 15 % caramel barley malt
5	BCBa	50% Blue corn malt, 35% base barley malt, 15% caramel barley malt
6	RCBa	50% Red corn malt, 35% base barley malt, 15% caramel barley malt

**Table 2:** Volatile compounds identified in all beer samples.

							Peak Area Counts × 10^6^				
No	Compound Name	LRI ^1^	LRI ^2^	BC	RC	RBC	Ba	BCBa	RCBa	ID ^3^	Flavour ^4^
**Alcohols**											
**1**	Ethanol	668	527	508.6 ± 132.5	510.5 ± 6.7	354.8 ± 107.4	672.2 ± 14.9	654.2 ± 18.7	548.3 ± 10.8	MS,S	Sweet ^6^
**2**	1-Propanol	536	605	8.8 ± 1.4	2.8 ± 1.6	6.1 ± 4.7	15.0 ± 10.5	6.5 ± 0.2	5.8 ± 0.1	MS,S	Sour ^6^
**3**	2-Methyl-1-propanol	647	644	16.2 ± 11.8	10.4 ± 0.2	16.2 ± 7.4	38.4 ± 28.6	18.1 ± 2.1	26.2 ± 6.7	MS,S	Wine, solvent, bitter ^6^
**4**	3-Methyl-1-butanol	736	750	237.9 ± 118.0	216.4 ± 40.0	171.2 ± 51.3	353.1 ± 22.1	164.7 ± 32.4	287.1 ± 85.8	MS	Whiskey, malt, burnt ^6^
**5**	2-Methyl-1-butanol	755	754	32.9 ± 13.3	32.8 ± 1.2	28.8 ± 9.7	76.6 ± 40.5	34.1 ± 4.4	60.5 ± 4.7	MS	Fermented ^6^
**6**	2,3-Butanediol	769	809	2.0 ± 0.3	2.4 ± 0.2	0.8 ± 0.3	2.32 ± 0.3	0.99 ± 0.2	0.2 ± 0	MS,S	Fruity ^6^
**7**	4-Methyl-1-pentanol	840	835	n.d	n.d	n.d	0.8 ± 0.6	0.3 ± 0.1	0.4 ± 0.1	MS	
**8**	2-Furanmethanol	851	846	n.d	n.d	n.d	5.7 ± 5.5	2 ± 1.8	1.1 ± 0.3	MS,S	Burnt ^6^
**9**	1-Hexanol	851	854	1.2 ± 1.3	2.4 ± 0.4	6.4 ± 1.8	5.8 ± 4.0	2.7 ± 0.2	3.8 ± 0.8	MS,S	Resin, flower, green ^6^
**10**	3-Methyl-1-hexanol	895	898	n.d	n.d	n.d	1.7 ± 0.8	0.5 ± 0.1	1.2 ± 0.3	MS	
**11**	1-Heptanol	962	986	1.3 ± 1.3	2.8 ± 1.1	3.2 ± 0.6	2.6 ± 1.7	2.6 ± 0.6	2.3 ± 0.7	MS,S	Chemical, green ^6^
**12**	1-Octen-3-ol	982	1004	n.d	n.d	1.3 ± 0.3	1.2 ± 0.6	1.2 ± 0.2	1.6 ± 0.2	MS,S	Mushroom, earthy ^6^
**13**	2-Ethyl-1-hexanol	1032	1054	3.3 ± 4.3	27.8 ± 3.2	38.7 ± 2.8	52.3 ± 22.0	43.8 ± 14.4	35.7 ± 11.6	MS	Rose, green ^6^
**14**	1-Octanol	1072	1096	5.4 ± 7.1	6.2 ± 2.3	4.7 ± 0.8	6.9 ± 3.6	10.4 ± 3.2	8.7 ± 2	MS,S	Chemical, metal ^6^
**15**	Phenylethyl alcohol	1118	1139	80.1 ± 72.9	36.6 ± 11.7	43.7 ± 5.1	154.4 ± 58.0	112.6 ± 67.7	112.5 ± 32.4	MS	Honey, spice, rose ^6^
**16**	(*Z*)-3-Nonen-1-ol	1152	1181	n.d	n.d	0.5 ± 0.001	0.8 ± 0.4	7.8 ± 2.1	1.5 ± 0.6	MS	Waxy, green, melon ^7^
**17**	1-Nonanol	1154	1198	n.d	2.0 ± 0.3	n.d	n.d	n.d	4.0 ± 1	MS	Fat, green ^6^
**18**	2-Decanol	1186	1229	14.3 ± 16.8	2.9 ± 2.8	n.d	n.d	n.d	n.d	MS	
**19**	Citronellol	1233	1255	19.6 ± 22.0	21.1 ± 9.7	3.6 ± 0.1	39.1 ± 20.2	16.8 ± 11.2	9.9 ± 3.1	MS,S	Rose ^6^
**20**	Iso-geraniol	1254	1262	n.d	n.d	n.d	2.0 ± 1.4	1.9 ± 1.7	1.0 ± 0.4	MS	Rose ^6^
**21**	1,9-Nonanediol	-	1292	n.d	1.7 ± 1.3	1.0 ± 0.001	n.d	0.9 ± 0.1	1.0 ± 0.4	MS	
**22**	1-Decanol	1263	1300	n.d	5.3 ± 3.5	2.2 ± 2.4	13.7 ± 8.6	5 ± 3.7	3.5 ± 1.2	MS	Fat ^6^
**23**	2-Undecanol	1294	1330	8.8 ± 9.1	2.2 ± 1.0	1.0 ± 0.3	5.7 ± 3.8	2.7 ± 1.7	1.5 ± 0.7	MS	
**24**	Caryophyllenyl alcohol	1568	1608	0.9 ± 0.5	0.3 ± 0.01	n.d	1.2 ± 0.8	n.d	n.d	MS	
**Aldehydes**											
**25**	Acetaldehyde	427	503	0.3 ± 0.3	n.d	n.d	0.5 ± 0.2	n.d	n.d	MS,S	Pungent, ether ^6^
**26**	Benzeneacetaldehyde	1044	1068	n.d	n.d	n.d	1.1 ± 0.3	0.5 ± 0.1	0.8 ± 0.2	MS	
**27**	Nonanal	1104	1130	3.7 ± 4.1	2.9 ± 1.9	1.5 ± 0.001	2.7 ± 1.1	5.4 ± 1.6	2.2 ± 0.8	MS	Fat, citrus, green ^6^
**28**	Decanal	1209	1233	8.2 ± 9.5	1.9 ± 1.7	2.7 ± 0.2	n.d	n.d	2.8 ± 1.6	MS	Soap, orange peel, tallow ^6^
**Aliphatic hydrocarbons**											
**29**	Tetradecane	1400	1430	2.2 ± 2.2	0.7 ± 0.3	1.2 ± 0.001	3.0 ± 1.4	n.d	n.d	MS	Waxy ^5^
**30**	Pentadecane	1500	1530	2.6 ± 2.9	0.6 ± 0.4	0.8 ± 0.4	3.0 ± 1.5	n.d	n.d	MS	Waxy ^5^
**Carboxylic acids**											
**31**	Acetic acid	600	619	11.7 ± 0.001	1.5 ± 0.5	n.d	n.d	1.2 ± 1.5	8.5 ± 1.2	MS,S	Sour ^6^
**32**	2-Methyl-propanoic acid	752	779	3.5 ± 0.7	2.4 ± 0.2	7.2 ± 2.3	3.1 ± 0.3	n.d	n.d	MS	Rancid butter ^5^
**33**	3-Methyl-butanoic acid	877	839	1.4 ± 0.001	1.8 ± 1.3	6.9 ± 5.3	3.4 ± 0.001	0.3 ± 0.1	0.8 ± 0.1	MS	Sweat, acid, rancid ^5^
**34**	2-Methyl-hexanoic acid	-	844	n.d	n.d	0.2 ± 0.1	n.d	1 ± 0.8	0.9 ± 0.5	MS	
**35**	2-Methyl-butanoic acid	896	845	n.d	n.d	0.5 ± 0.1	1.3 ± 0.2	0.7 ± 0.3	n.d	MS	
**36**	Hexanoic acid	1019	1010	n.d	2.2 ± 0.1	21.4 ± 4.4	17.8 ± 0.2	n.d	13.0 ± 0.6	MS,S	Fatty, sour, sweat, cheese ^6^
**37**	Heptanoic acid	1078	1103	n.d	1.5 ± 1.6	n.d	2.6 ± 2.4	1.0 ± 0.4	1.3 ± 0.7	MS	Cheesy, waxy, sweaty ^5^
**38**	2-Ethyl-hexanoic acid	1116	1167	0.9 ± 0.8	1.0 ± 0.5	n.d	3.8 ± 1.3	n.d	0.6 ± 0.9	MS	
**39**	Octanoic acid	1279	1209	608.8 ± 5.3	194.3 ± 4.5	217.3 ± 1.6	411.8 ± 16.4	12 ± 0.4	458.1 ± 30.7	MS,S	Sweat, cheese ^6^
**40**	9-Decenoic acid	1358	1392	35.8 ± 31.8	8.6 ± 6.8	10.9 ± 0.9	n.d	7.4 ± 0.6	n.d	MS	
**41**	Decanoic acid	1373	1399	51.0 ± 45.7	n.d	23.5 ± 7.0	68.0 ± 54.7	7.8 ± 0.5	10.9 ± 2.9	MS,S	Rancid, fat ^6^
**42**	Hexadecanoic acid	1984	2000	n.d	3.8 ± 4.0	3.3 ± 3.9	1.6 ± 1.7	n.d	n.d	MS,S	Oily ^6^
**Esters**											
**43**	Ethyl acetate	628	635	25.0 ± 12.7	16.6 ± 9.6	27.1 ± 1.9	57.7 ± 34.8	42.5 ± 6.5	46.0 ± 16.3	MS,S	Pineapple ^6^
**44**	Ethyl propanoate	713	728	n.d	n.d	2.1 ± 1.6	2.7 ± 1.4	2 ± 0.1	2.4 ± 0.3	MS	Fruit ^6^
**45**	Propyl acetate	720	731	n.d	n.d	n.d	1.0 ± 0.2	n.d	0.4 ± 0.1	MS	Sweet, fruity, caramel ^7^
**46**	Ethyl isobutanoate	756	780	n.d	n.d	n.d	1.3 ± 0.4	1.2 ± 0.1	2.8 ± 0.8	MS	Sweet, rubber ^6^
**47**	Isobutyl acetate	776	798	1.8 ± 0.2	1.4 ± 0.3	1.7 ± 1.3	1.4 ± 0.7	2.3 ± 0.2	2.3 ± 0.8	MS	Fruit, apple, banana ^6^
**48**	Ethyl butanoate	804	814	2.2 ± 0.3	2.1 ± 0.5	1.9 ± 0.1	4.5 ± 2.7	9 ± 0.3	5.8 ± 1.8	MS	Apple ^6^
**49**	3-Methylbutyl acetate	877	859	31.7 ± 18.5	38.7 ± 12.7	20.3 ± 7.9	47.9 ± 25.6	113 ± 2.6	66.6 ± 19.7	MS	Fresh, banana, sweet ^5^
**50**	2-Methylbutyl acetate	876	861	2.0 ± 1.2	3.0 ± 1.1	1.2 ± 0.7	2.7 ± 1.5	5.2 ± 0.7	5.6 ± 1.5	MS	Herbal, fermented fruity ^5^
**51**	Ethyl pentanoate	900	875	n.d	n.d	n.d	1.1 ± 0.6	1.5 ± 0.1	2.2 ± 0.5	MS	Yeast, fruit ^7^
**52**	Ethyl iso-hexanoate	-	974	n.d	n.d	n.d	0.8 ± 0.4	1.3 ± 0.7	1.4 ± 0.5	MS,T	Sweet, fruity, tropical, green, apple ^7^
**53**	Methylbutyl propanoate	-	992	0.7 ± 0.5	1.2 ± 0.8	n.d	n.d	0.9 ± 0.4	n.d	MS,T	
**54**	Ethyl hexanoate	1002	1025	8.9 ± 7.5	14.8 ± 8.8	10.0 ± 2.2	27.7 ± 13.9	151.2 ± 89.3	139.8 ± 41.5	MS,S	Apple peel, fruit ^6^
**55**	Hexyl acetate	1014	1039	2.1 ± 1.4	3.6 ± 2.3	1.8 ± 0.6	1.8 ± 0.4	3.3 ± 1.2	1.3 ± 0.4	MS	Fruity, spicy, herbal, sweet wine, rubbery ^7^
**56**	2-Metylbutyl isobutanoate	1014	1042	5.0 ± 2.9	15.7 ± 10.2	n.d	n.d	1.1 ± 0.4	n.d	MS	Fruity, ethereal ^7^
**57**	Ethyl 5-methylhexanoate	-	1088	n.d	1.1 ± 0.5	n.d	0.9 ± 0.3	6.7 ± 3.5	4.3 ± 1.2	MS,T	
**58**	Ethyl benzoate	1185	1197	n.d	n.d	n.d	4.0 ± 2.2	2.7± 1.7	n.d	MS	Chamomile, flower ^6^
**59**	Ethyl octanoate	1198	1225	67.1 ± 59.9	63.5 ± 36.1	51.8 ± 6.2	n.d	724.7 ± 45.0	3.9 ± 1.3	MS,S	Fruit, fat ^6^
**60**	Ethyl phenylacetate	1252	1273	n.d	n.d	0.8 ± 0.2	2.8 ± 1.3	2.2 ± 1.3	1.4 ± 0.7	MS	Fruit, sweet ^7^
**61**	Phenethyl acetate	1265	1285	14.5 ± 13.9	5.4 ± 2.4	5.8 ± 0.8	20.6 ± 9.3	15.2 ± 8.3	8.5 ± 2.8	MS	Rose, floral ^7^
**62**	Ethyl nonanoate	1295	1326	1.8 ± 1.5	n.d	0.7 ± 0.00	2.0 ± 1.1	3.2 ± 1.9	1.9 ± 0.9	MS	Fruity, rose ^6^
**63**	Methyl geranoate	1323	1354	6.6 ± 6.1	3.0 ± 1.6	3.3 ± 0.7	3.4 ± 1.6	2.2 ±1.5	0.7 ± 0.3	MS	Floral ^6^
**64**	Ethyl benzenepropanoate	1390	1379	n.d	n.d	n.d	1.7 ± 1.0	1.5 ± 1	n.d	MS	
**65**	Ethyl (*E*)-4-decenoate	-	1408	4.3 ± 3.0	1.7 ± 0.8	n.d	6.9 ± 1.0	3.6 ±1.2	3.7 ± 1.1	MS,T	
**66**	Ethyl 9-decenoate	1387	1417	15.2 ± 1.2	13.2 ± 7.0	12.7 ± 3.2	15.5 ± 6.3	59.6 ± 18.7	22.7 ± 6.4	MS	
**67**	Ethyl decanoate	1397	1426	20.3 ± 14.0	12.5 ± 5.9	13.5 ± 4.6	50.1 ± 17.1	24.3 ± 5.9	31.6 ± 10.6	MS,S	Grape, fruit ^6^
**68**	Isoamyl octanoate	-	1478	0.7 ± 0.6	n.d	0.3 ± 0.1	1.5 ± 0.5	0.7 ± 0.3	1.1 ± 0.4	MS,T	
**69**	Ethyl dodecanoate	1494	1628	9.9 ± 4.3	3.1 ± 1.3	1.6 ± 0.7	8.7 ± 4.4	n.d	n.d	MS	Leaf ^6^
**70**	Dibutyl maleate	-	1571	1.6 ± 1.1	n.d	0.4 ± 0.001	0.8 ± 0.6	n.d	n.d	MS,T	
**71**	Ethyl *cis*-9-pentadecenoate	-	1622	6.4 ± 4.3	0.9 ± 0.5	0.4 ± 0.1	n.d	n.d	n.d	MS,T	
**72**	Ethyl tetradecanoate	1793	1832	1.5 ± 0.6	0.7 ± 0.1	0.5 ± 0.1	1.7 ± 1.0	4.8 ± 1.6	n.d	MS,S	Oily, violet ^6^
**73**	2-Ethylhexyl salicylate	1816	1847	1.3 ± 0.7	2.9 ± 3.7	1.6 ± 0.5	5.2 ± 6.4	0.9 ± 0.5	1.2 ± 0	MS	
**74**	Ethyl 9-hexadecenoate	-	2015	n.d	0.9 ± 0.5	1.1 ± 1.0	0.9 ± 0.6	0.3 ± 0.1	n.d	MS,T	
**75**	Ethyl hexadecanoate	1991	2038	1.1 ± 0.2	1.1 ± 0.4	1.9 ± 0.9	2.1 ± 1.5	0.3 ± 0.2	n.d	MS	Waxy ^6^
**76**	Isopropyl palmitate	-	2070	n.d	n.d	2.5 ± 2.5	2.2 ±2.6	n.d	n.d	MS,T	
**77**	1-Propylpentyl dodecanoate	-	2152	0.6 ± 0.2	3.5 ± 3.9	2.8 ± 2.5	4.5 ± 5.2	n.d	n.d	MS,T	
**Furans**											
**78**	Acetylfuran	893	881	n.d	n.d	n.d	1.8 ± 1.5	0.3 ± 0.1	n.d	MS,S	Balsamic ^6^
**79**	3-Methyl-2,3-dihydro-1-benzofuran	-	1178	n.d	n.d	0.5 ± 0.1	n.d	1.3 ± 1.0	n.d	MS,T	
**80**	2,3-Dihydro-benzofuran	-	1246	2.5 ± 2.7	3.9 ± 0.5	n.d	4.3 ± 2.0	n.d	3.9 ± 1.1	MS,T	
**81**	Dihydro-5-pentyl-2(3H)-furanone	-	1392	n.d	n.d	n.d	16.7 ± 7.7	5.6 ± 1.6	7.8 ± 2.8	MS,T	
**Aromatic hydrocarbons**											
**82**	Styrene	893	867	33.2 ± 18.7	32.3 ± 19.7	25.7 ± 0.4	56.8 ± 25.3	52.9 ± 13.1	78.0 ± 27.4	MS	Balsamic, gasoline ^6^
**83**	1,4-Dichloro-benzene	1015	1035	29.0 ± 28.7	13.0 ± 0.5	23.5 ± 3.3	29.7 ± 11.4	18.0 ± 1.1	15.7 ± 2.7	MS	Mothball-like ^5^
**84**	Squalene	2833	2881	2.6 ± 0.3	16.4 ± 16.2	18.7 ± 21.0	12.9 ± 14.8	n.d	n.d	MS	
**Ketones**											
**85**	2-Pentanone	636	708	n.d	n.d	n.d	0.6 ± 0.4	0.6 ± 0	n.d	MS	Ether ^6^
**86**	3-Methyl-2-pentanone	759	777	n.d	1.1 ± 1.2	n.d	n.d	n.d	n.d	MS	
**87**	Acetophenone	1041	1091	n.d	n.d	0.4 ± 0.1	0.7 ± 0.2	n.d	n.d	MS,S	Must, flower, almond ^6^
**88**	2-Nonanone	1091	1118	n.d	1.3 ± 0.8	n.d	n.d	0.9 ± 0.3	n.d	MS	Fruity, sweet, waxy, soapy, herbaceous, coconut ^5^
**89**	β-Damascenone	1386	1386	n.d	n.d	n.d	n.d	7.8 ± 2.7	6.7 ± 1.6	MS	
**90**	β-Ionone	1493	1526	1.2 ± 0.1	0.8 ± 0.2	n.d	n.d	0.6 ± 0.3	0.4 ± 0.2	MS,S	Seaweed, violet, flower, raspberry ^6^
**Miscellaneous**											
**91**	Methoxy-phenyl-oxime	-	883	9.1 ± 6.9	4.1 ± 1.3	3.5 ± 0.4	8.2 ± 7.4	n.d	0.5 ± 0.3	MS,T	
**92**	Geranyl vinyl ether	-	1259	3.0 ± 3.0	1.3 ± 1.0	n.d	n.d	n.d	n.d	MS,T	
**93**	9-Decen-1-ol methyl ether	-	1312	7.5 ± 7.8	n.d	0.7 ± 0.2	n.d	1.6 ± 0.01	n.d	MS,T	
**Phenols**											
**94**	Phenol	980	1007	n.d	n.d	5.7 ± 0.001	4.6 ± 1.5	n.d	1.0 ± 0.5	MS	Phenolic, medicinal ^6^
**95**	2-Methoxy-phenol	1089	1115	3.3 ± 3.6	n.d	2.3 ± 0.3	n.d	2.4 ± 1.9	0.4 ± 0.1	MS,S	Smoke, sweet, medicine ^6^
**96**	4-Ethyl-phenol	1287	1193	4.4 ± 4.9	0.8 ± 0.1	41.4 ± 4.0	1.5 ± 0.1	0.7 ± 0.3	1.0 ± 0.4	MS,S	Spice, clove ^6^
**97**	4-Ethyl-2-methoxy-phenol	-	1308	8.0 ± 7.9	1.7 ± 0.8	22.2 ± 4.0	n.d	2.3 ± 2	2.0 ± 0.8	MS,T	Spice, smoke, clove, medicinal ^5^
**98**	2-Methyl-5-(1-methylethyl)-phenol	1307	1323	4.4 ± 4.9	0.7 ± 0.5	0.5 ± 0.2	2.0 ± 1.8	n.d	n.d	MS	Spicy, cooling, thymol-like, herbal and camphoreous ^5^
**99**	2-Methoxy-4-vinylphenol	1315	1344	1.8 ± 1.6	21.2 ± 4.7	23.0 ± 4.2	15.4 ± 7.8	2.5 ± 1.5	2.6 ± 0.6	MS	Smoky, bacon ^5^
**100**	2,6-Di-tert-butylphenol	1444	1502	2.0 ± 2.0	n.d	2.8 ± 0.001	n.d	n.d	n.d	MS,T	Phenolic ^5^
**Pyrrole and pyrazine**											
**101**	2-Acetylpyrrole	1045	1086	n.d	n.d	n.d	1.3 ± 0.5	0.5 ± 0.2	n.d	MS	Nut, walnut, bread ^6^
**102**	Tetramethyl-pyrazine	-	1122	n.d	n.d	n.d	1.2 ± 0.3	1.6 ± 0.6	0.7 ± 0.1	MS,T	Nutty ^7^
**Sulphur compounds**											
**103**	Dimethyl sulfide	505	569	3.8 ± 2.6	3.2 ± 1.6	3.8 ± 0.4	4.7 ± 1.2	2.7 ± 0.4	2.4 ± 0	MS	Cabbage, sulphur, gasoline ^6^
**Terpenes**											
**104**	β-Myrcene	992	1016	10.7 ± 11.9	21.9 ± 17.8	n.d	n.d	4.7 ± 1.7	9.6 ± 4.7	MS,S	Balsamic, must, spice ^6^
**105**	Limonene	1033	1056	42.6 ± 57.6	11.3 ± 5.8	1.2 ± 0.5	0.7 ± 0.5	n.d	n.d	MS,S	Lemon, orange ^6^
**106**	Linalool	1100	1126	40.7 ± 46.9	37.0 ± 14.1	8.7 ± 0.4	22.3 ± 9.9	28.3 ± 11.9	18.5 ± 4.8	MS,S	Flower, lavender ^6^
**107**	Camphor	1139	1171	n.d	n.d	0.6 ± 0.1	1.3 ± 0.8	1 ± 0.9	n.d	MS,S	Camphor ^6^
**108**	Geraniol	1276	1283	12.0 ± 13.1	3.8 ± 2.1	2.2 ± 0.2	13.8 ± 7.9	3.6 ± 2.2	1.9 ± 0.8	MS,S	Rose, geranium ^6^
**109**	Caryophyllene	1467	1454	2.4 ± 1.8	1.5 ± 0.4	n.d	1.5 ± 0.4	0.8 ± 0.2	2.7 ± 1	MS,S	Wood, spice ^6^
**110**	Humulene	1467	1489	11.3 ± 5.1	7.4 ± 2.5	0.7 ± 0.1	6.5 ± 2.2	1 ± 0.4	11.6 ± 4.5	MS,S	Wood ^6^
**111**	3-Methoxy-2-naphthalenol	-	1518	n.d	n.d	n.d	2.7 ± 1.3	0.3 ± 0.2	0.4 ± 0.2	MS,T	
**112**	δ-Cadinene	1519	1559	0.7 ± 0.3	n.d	n.d	1.2 ± 0.3	n.d	0.5 ± 0.2	MS	Thyme, medicine, wood ^6^
**113**	*E*-Nerolidol	1539	1597	2.7 ± 1.8	n.d	0.6 ± 0.1	3.6 ± 1.8	1.2 ± 0.6	0.5 ± 0.2	MS	Wood, flower, wax ^6^
**114**	Caryophyllene oxide	1573	1612	1.4 ± 1.3	n.d	n.d	1.7 ± 1.1	0.8 ± 0.4	0.7 ± 0.1	MS,T	Herb, sweet, spice ^6^
**115**	Humulene oxide	1642	1641	1.3 ± 1.0	0.9 ± 0.3	0.3 ± 0.1	9.8 ± 5.3	0.4 ± 0.2	n.d	MS,S	Herb ^6^
**116**	Cubenol	1645	1666	1.2 ± 0.7	0.9 ± 0.5	0.4 ± 0.2	1.2 ± 0.4	0.4 ± 0.2	n.d	MS	Spice, herb, green tea ^6^
**117**	Di-epi-1,10-cubenol	1613	1669	2.0 ± 1.2	n.d	n.d	3.3 ± 1.7	0.3 ± 0.1	n.d	MS	
**118**	Calarene epoxide	-	1672	7.9 ± 4.9	n.d	n.d	n.d	1.1 ± 0.6	0.7 ± 0.1	MS,T	Woody ^5^
**119**	τ-Cadinol	1635	1679	4.3 ± 2.8	n.d	n.d	4.5 ± 3.0	n.d	n.d	MS	Herb, weak spice ^6^
**120**	δ-Cadinol	1674	1689	0.5 ± 0.6	n.d	n.d	1.1 ± 0.1	0.7 ± 0.3	n.d	MS	Herb ^6^
**121**	α-Cadinol	1676	1695	2.8 ± 1.8	n.d	n.d	1.9 ± 1.9	0.3 ± 0.1	n.d	MS	Herb, wood ^6^

Chromatographic peak area (peak area counts × 10^6^) of the flavour volatile compounds. Results are expressed as mean ± standard deviation (n = 2); BC = 100% blue corn, RC = 100% red corn, RBC = 50:50 red and blue corn, Ba = 100% barley, BCBa = 50:50 blue corn and barley, RCBa = 50:50 red corn and barley. ^1^ LRI = Linear retention index (NIST values (http://webbook.nist.gov/chemistry/name-ser.html). ^2^ LRI = Linear retention index on HP-5MS column (Agilent Technologies), calculated via duplicated averaged alkanes, and found to be comparable with NIST values (http://webbook.nist.gov/chemistry/name-ser.html). ^3^ ID = Identification used as confirmation of compounds per: MS = library match; S = standards; T = tentative. ^4^ Flavour descriptors according to ^5^ The Good Scents Company (http://www.thegoodscentscompany.com/), ^6^ Flavornet (http://www.flavornet.org/flavornet.html) and ^7^ Pherobase (http://www.pherobase.com/). n.d. no detected.

**Table 3:** Sensory attributes, description and physical references used in this study (attributes pertaining to appearance, odour, aroma, taste and mouthfeel are designated by an “Ap”, “O”, “A”, “T”, “M” respectively after the attribute).

Attributes	Abbreviation	Description	Reference
Colour	Colour-Ap	Refers to the colour of the beer	Standard Reference Method (SRM) scale
Turbidity	Turbidity-Ap	Refers to the haziness of the beer	Range of different beer samples
Banana	Banana-O	Sweet gum flavoured banana	Isoamyl acetate (Siebel^®^ kit)
Fruity	Fruity-O	A mix of fruits as pear, strawberry and grapefruit	Linalool (Siebel^®^ kit)
Apple	Apple-O	Green apple	Acetaldehyde (Siebel^®^ kit)
Cooked corn	Cook.corn-O	“Esquites odour”	Dimethyl sulfide (Siebel^®^ kit)
Fermented fruits	Ferm fruits-O	Traditional fermented beverage made of a mix of fruits as pineapple, guava and apple	“Tepache”
Dried fruits	Dried fruits-O	Raisins, prunes, plum	Firmenich^®^ reference
Dried chili	Dried chili-O	Odour of the Guajillo chili	Guajillo chili (6 g/L)
Pineapple	Pineapple-O	Ripe pineapple	Firmenich^®^ reference
Hoppy	Hoppy-O	Pine -herbaceous odour	Tea made of Saaz and Magnum hops (0.5 g/L)
Bread	Bread-O	Fresh bread recently cooked	Firmenich^®^ reference
Caramel	Caramel-O	Associated to caramel	Firmenich^®^ reference
Brown sugar	Brown sugar-O	Product elaborated from raw brown sugar	“Piloncillo”
Olive	Olive-O	Vinegar-like	Acetic acid (Siebel^®^ kit)
Floral	Floral-O	Flowers-like, roses	Geraniol (Siebel^®^ kit)
Hoppy	Hoppy.A	Pine -herbaceous aroma	Tea made of Saaz and Magnum hops (0.5 g/L)
Malty	Malty.A	Malty-like	Firmenich^®^ reference
Alcohol	Alcohol-A	A warming sensation in the mouth and throat	Firmenich^®^ reference
Cooked vegetables	Cook.veg-A	Mix of cooked vegetables	Dimethyl sulfide (Siebel^®^ kit)
Burnt tortillas	Tortillas-A	Aroma related to tortillas after being heated	Burnt tortillas
Sweet	Sweet-T	Associate with sugar taste	Sucrose 7.5 g/L
Bitter	Bitter-T	Associate with bitter taste	Isolone (Siebel^®^ kit)
Sour	Sour-T	Associate with acid taste	Lactic acid (Siebel^®^ kit)
Oxidised	Oxidised-M	Papery, cardboard	trans-2-nonenal (Siebel^®^ kit)
Spicy	Spicy-M	Pungent sensation in the tongue caused by chili	Guajillo chili (6 g/L)
Metallic	Metallic-M	Metal-like	Ferrous sulfate (Siebel^®^ kit)
Astringent	Astringent-M	Sensation of dryness in the tongue and mouth	Tannic acid (0.6 g/L)
Carbonatation	Carbonatation-M	Sensation tingle in the tongue related to CO_2_	Peñafiel mineral water
Fullness	Fullness-M	Refers to the perceived density while it is being consumed	Range of different beer samples

**Table 4:** Mean score of beer samples for each non-volatile parameter.

Parameter		Beer					
		BC	RC	RBC	Ba	BCBa	RCBa
Alcohol (%, v/v)	ABV	3.71 c	2.98 cd	1.93 d	7.01 ab	5.45 b	7.21 a
International bitterness units	IBU	14.57 b	19.05 a	19.62 a	18.45 a	18.92 a	15.72 b
Anthocyanins (mg/L)	TAC	14.45 a	8.84 b	14.60 a	0.00 e	3.90 d	6.17 c
Polyphenols (mg GAE/L)	TPC	750.0 ab	331.0 c	367.5 c	398.5 c	849.5 a	721 b

Values with different letters across a row are significantly different (*p* < 0.05) according to the Tukey post-hoc test. BC = 100% blue corn, RC = 100% red corn, RBC = 50:50 red and blue corn, Ba = 100% barley, BCBa = 50:50 blue corn and barley, RCBa = 50:50 red corn and barley.

**Table 5:** RV coefficients between odour-aroma, taste-mouthfeel, volatiles, non-volatiles and supplementary data matrices of the MFA.

	Odour-Aroma	Taste-Mouthfeel	Volatiles	Non-Volatiles	Supplementary	MFA
Odour-Aroma	1.000	0.403	0.509	0.740	0.741	0.846
Taste-mouthfeel	0.403	1.000	0.649	0.374	0.307	0.755
Volatiles	0.509	0.649	1.000	0.364	0.428	0.793
Non-volatiles	0.740	0.374	0.364	1.000	0.321	0.779
Supplementary	0.741	0.307	0.428	0.321	1.000	0.579
MFA	0.846	0.755	0.793	0.779	0.579	1.000

## Supplementary Materials

The following are available online at https://www.mdpi.com/2304-8158/9/7/886/s1, Table S1: Sensory attribute score means of beer samples.
