# Supplementary material for: Renewing Traditions: A Sensory and Chemical Characterisation of Mexican Pigmented Corn Beers
Source: Foods. 2020 Jul 6;9(7):886. doi: 10.3390/foods9070886 (PMC7404799; doi:10.3390/foods9070886)
Supplement: Supplementary file 1 [file foods-09-00886-s001.pdf]

SUPPLEMENTARY INFORMATION FOR
Renewing Traditions: a Sensory and Chemical Characterisation of Mexican Pigmented Corn Beers

TABLE OF CONTENTS

Table S1. Sensory attribute score means of beer samples.

Attributes	Beers					
	**BC**	**RC**	**RBC**	**Ba**	**BCBa**	**RCBa**
**Colour-Ap**	15.0 c	9.00 a	12.0 b	13.0 bc	12.0 b	13.0 b
**Turbidity-Ap**	8.17 bc	4.18 a	8.89 bc	7.49 b	9.67 c	10.02 c
**Banana-O**	2.33 a	3.15 a	2.68 a	3.32 a	2.52 a	3.13 a
**Fruity-O**	3.15 a	3.04 a	2.93 a	3.25 a	2.96 a	4.27 a
**Apple-O**	2.60 a	2.57 a	1.64 a	3.02 a	2.64 a	3.03 a
**Cook corn-O**	2.37 a	3.33 a	2.86 a	1.91 a	2.54 a	2.12 a
**Ferm fruits-O**	2.04 a	3.67 a	3.57 a	2.60 a	2.28 a	2.41 a
**Dried fruits-O**	2.50 a	2.44 a	3.13 a	2.56 a	2.79 a	2.23 a
**Dried chili-O**	2.73 a	2.74 a	3.28 a	2.25 a	3.26 a	2.81 a
**Pineapple-O**	2.74 a	2.84 a	2.78 a	3.23 a	2.48 a	2.90 a
**Hoppy-O**	2.92 ab	3.28 ab	2.47 a	2.95 ab	2.24 a	3.88 b
**Bread-O**	1.11 a	1.51 a	1.37 a	0.94 a	1.35 a	1.07 a
**Caramel-O**	2.71 ab	1.99 a	2.40 ab	3.59 ab	3.97 b	3.02 ab
**Brown sugar-O**	2.39 a	2.19 a	1.98 a	3.66 a	3.31 a	2.96 a
**Olive-O**	0.92 a	1.80 a	1.51 a	0.85 a	1.03 a	1.15 a
**Floral-O**	2.36 a	2.77 a	2.33 a	2.85 a	1.78 a	2.86 a
**Sweet-T**	2.88 a	2.32 a	3.29 a	3.32 a	2.86 a	2.63 a
**Bitter-T**	2.46 a	5.51 bc	3.99 ab	4.26 ab	3.27 a	6.73 c
**Oxidised-T**	1.97 a	5.03 b	3.53 ab	3.04 a	2.89 a	3.36 ab
**Sour-T**	0.94 a	3.67 b	1.58 a	2.24 ab	2.46 ab	1.95 ab
**Hoppy-A**	2.68 a	4.51 bc	3.58 ab	3.78 ab	3.17 ab	5.94 c
**Malty-A**	3.26 ab	3.89 ab	2.83 a	3.99 ab	3.59 ab	4.92 b
**Alcohol-A**	2.67 a	2.64 a	2.80 a	4.22 b	3.61 ab	3.22 ab
**Cook vege-A**	1.59 a	3.39 b	2.21 ab	1.48 a	1.71 a	1.43 a
**Tortillas-A**	1.57 a	3.07 b	1.63 a	1.75 ab	2.04 ab	2.87 ab
**Spicy-M**	5.74 b	3.32 a	3.13 a	4.56 ab	7.84 c	3.20 a
**Metallic-M**	2.07 a	5.71 c	4.34 bc	2.56 a	2.45 a	2.85 ab
**Astringent-M**	2.23 a	2.88 a	3.10 a	3.73 a	2.58 a	3.13 a
**Carbonatation-M**	4.32 ab	2.88 a	2.85 a	6.17 b	3.70 a	4.92 ab
**Fullness-M**	6.08 ab	4.24 a	4.95 a	7.30 b	5.86 ab	7.78 b

The data are the average of the two replicates. Values with different letters across a row are significantly different ($p<0.05$) according to the Tukey's post-hoc test. Colour-Ap in Standard Reference Method (SRM) values.
